# Atomic Force Microscopy of Phytosterol Based Edible Oleogels

**DOI:** 10.3390/gels9090750

**Published:** 2023-09-15

**Authors:** Andrew B. Matheson, Vasileios Koutsos, Stephen R. Euston, Paul S. Clegg

**Affiliations:** 1 School of Physics and Astronomy, University of Edinburgh, James Clerk Maxwell Building, Edinburgh EH9 3FD, UK; 2 School of Engineering, Institute for Materials and Processes, University of Edinburgh, Sanderson Building, Edinburgh EH9 3FB, UK; 3 Institute of Biological Chemistry, Biophysics and Bioengineering, Heriot-Watt University, Edinburgh EH14 4AS, UK; 4 Department of Physics, Toronto Metropolitan University, Toronto, ON M5B 0C3, Canada

**Keywords:** oleogels, atomic force microscopy, triglycerides, phytosterols

## Abstract

This work reviews the use of atomic force microscopy (AFM) as a tool to investigate oleogels of edible triglyceride oils. Specific attention is given to those oleogels based on phytosterols and their esters, a class of material the authors have studied extensively. This work consists of a summary of the role of AFM in imaging edible oleogels, including the processing and preparation steps required to obtain high-quality AFM images of them. Finally, there is a comparison between AFM and other techniques that may be used to obtain structural information from oleogel samples. The aim of this review is to provide a useful introduction and summary of the technique for researchers in the fields of gels and food sciences looking to perform AFM measurements on edible oleogels.

## 1. Introduction

An oleogel is a liquid oil that has been gelled through the addition of a material or materials that form a network that arrests fluid flow, imbuing the oil with viscoelastic or solid-like properties. Oleogels have many possible applications, including in 3D printing [1] and drug delivery [2], and most prominently in food processing, where gelled triglyceride oils could be used to replace saturated fats [3,4,5].

The mechanism for gelation depends on the specific molecules being used to form the gel, known as the gelator or structurant. Gels may be formed from fibres, crystals, or droplets, which link together to create a network throughout the continuous oil phase of the oleogel, and there are various food-safe molecules that exhibit the necessary behaviour to gel oils. These broadly fall into three classes: polymers [6], fatty acids and alcohols [7], and other bio-derived small molecules [8]. In polymer-based oleogels, the gel network is formed from entangled polymer chains, whereas, in small-molecule oleogels, the gelator molecules must self-assemble into the necessary structures required to gel the oil. One of the most interesting families of small-molecule oleogators are phytosterols [4,9], a class of plant-derived small molecules of the same family as cholesterol that exhibit complex self-assembly behaviour to form fluid spanning networks in oil [8]. 

Understanding the complex structures of these gels is key to optimising processing steps for possible commercial applications, and, to this end, atomic force microscopy (AFM) has proven to be a very powerful tool [9,10,11,12,13,14]. AFM is a scanning probe microscopy technique whereby a sharp probe tip, mounted at the end of a cantilever, is rastered over the material surface that is to be imaged, and the deflection of a laser beam reflected off the end of the cantilever is used to monitor the position of the tip and build a picture of the topography and material properties of that surface. 

The number of triglyceride oleogel systems that have been imaged using AFM is relatively low. The authors of this review have extensively observed phytosterol oleogels [9,10], as well as gels based on synthetically derived sterols and lanosterol [11]. These gels typically all consist of self-assembled fibrils of ~10 nm thickness, which then form larger fibres that enmesh to form the fluid spanning network. This group was not the first to perform AFM on such gels [15], and, since then, several other researchers have used AFM to explore how changes in the solvent phase may alter gel formation [12,13,14]. The other family of triglyceride gels that has been studied in great detail with AFM are ethyl-cellulose-based gels [16,17,18]. Several other AFM studies have been carried out on triglyceride systems, including, but not exclusive to, gels using monoglycerides of fatty acids or fatty alcohols [7], xanthan [19], and wax crystals [20].

As with any advanced imaging technique, there are many nuances and subtleties to obtaining AFM images successfully. In this paper, we outline the use of AFM as a tool for imaging edible oleogels and then briefly compare and contrast AFM to other techniques that may be used to provide structural information. This article should aid other researchers looking to perform AFM measurements on this fascinating class of systems.

## 2. An Overview of AFM Imaging of Oleogels in Triglyceride Oils

**Figure 1:**
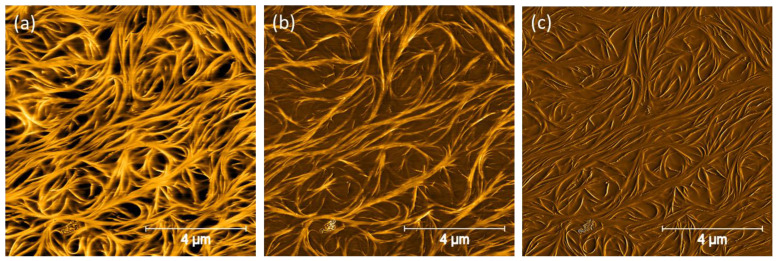
AFM height: (**a**) phase, (**b**), and amplitude (**c**) images obtained simultaneously for a sitosterol–oryzanol oleogel. Reprinted (adapted) with permission from Langmuir 2017, 33, 18, 4537–4542. Copyright 2017 American Chemical Society.

The most common way to operate an AFM when imaging oleogels is in tapping mode (TM). This is the specific AFM modality whereby the cantilever is driven to oscillate close to its resonant frequency, contacting the sample intermittently; the sample height is adjusted by the use of a piezoelectric scanner and a feedback control loop to maintain the cantilever oscillation amplitude constant. This height information combined with raster scanning provides a map of the topography of the system, as shown in Figure 1a. As well as the height, the phase and amplitude of the oscillations are also recorded. The phase (Figure 1b) is particularly useful as it is primarily altered by the stiffness and/or stickiness of the region beneath the tip. Therefore, the phase map provides a qualitative indication of contrasting material properties or concentrations across the sample and may reveal details absent from the height map. Finally, the amplitude image can reveal subtle surface structures because it enhances the appearance of abrupt changes in height, such as edges and boundaries, as shown in Figure 1c. As can be seen by comparing Figure 1a–c, although they are recognisably images of the same field of view, different aspects of the sample are highlighted by each imaging modality. This ability to obtain three images with differing contrast makes AFM an appealing choice for imaging oleogels as, even if topography is hard to discern, there should be significant differences in the mechanical properties of the structurant and oil phase.

**Figure 2:**
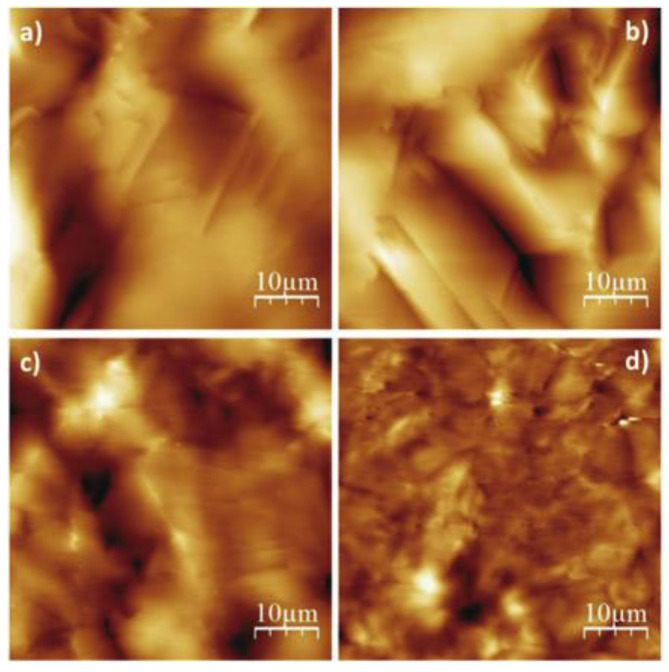
AFM images of oleogels based upon monoglycerides structuring: (**a**) sunflower oil; (**b**) rice oil; (**c**) paraffin oil; (**d**) castor oil. Reprinted from Food Research International, 111, Lupi et al., The role of edible oils in low molecular weight organogels rheology and structure, 399–407., Copyright (2018), with permission from Elsevier.

However, oleogels based on edible oils such as triglycerides present a particularly challenging class of materials to image using AFM. AFM systems are generally used to image dried or (through the use of a specialised water cell) aqueous samples. It is very difficult to dry a triglyceride edible oleogel (in the way one may an oleogel produced from a more volatile solvent [21]) due to the very low vapour pressure of the triglyceride oil. There have been several AFM studies of oleogels based on triglycerides, employing a range of approaches to sample preparation. This includes work where oleogels have been cast directly onto substrates and images taken without any additional processing steps. Such an approach was taken by Lupi et al. when obtaining the images shown in Figure 2, which was adequate to reveal structures on the tens of micron length scale of the crystalline domains of fatty alcohols or acids [7]. However, to image gels on a “nano” scale, additional steps are generally needed.

The other extreme is to remove all the oil from the sample. One technique for doing so is to cast the gel on the substrate and then wait for it to set before leaving the sample to soak in isobutanol for 24 h and then air drying. This effectively removes the liquid phase from the gel completely, leaving only a skeleton or xerogel. The sample can then be imaged suspended in water using a liquid cell AFM. This approach has proven to be highly effective in the imaging of oleogel structures based on the polymer ethyl cellulose [16,17,22]. However, these steps may not be appropriate for all gel structures where the gel network may not be strong enough to survive the process of solvent exchange and may not be stable in either the isobutanol or in water. The β-sitosterol and γ-oryzanol gels fall into this category due to the tendency for β-sitosterol to form hydrogen bonds with water, leading to it being thermodynamically stabilised out of the mixture in the form of a crystal hydrate [9,23,24].

**Figure 3:**
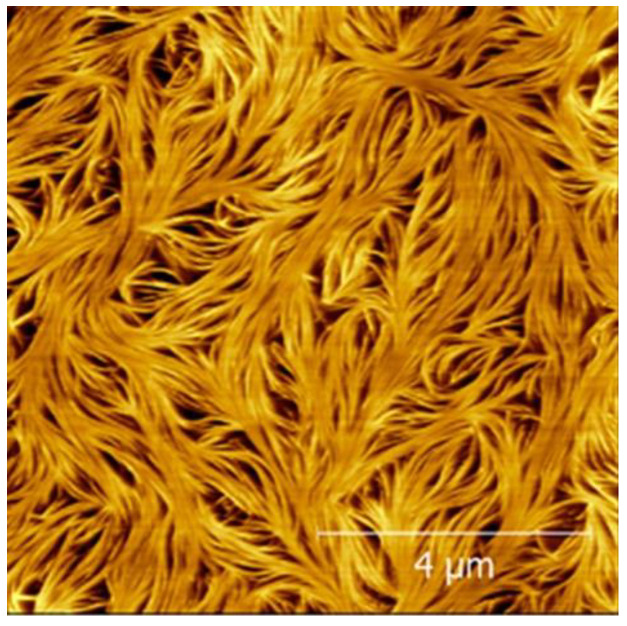
AFM height image of sitosterol and oryzanol dipcast onto mica. Reprinted (adapted) with permission from Langmuir 2017, 33, 18, 4537–4542. Copyright (2017) American Chemical Society.

However, as AFM is inherently a probe of the surface, there is no need to remove oil from the entire bulk of the sample. Sawalha et al. outlined an alternative approach, whereby, rather than totally removing all triglyceride from the sample, the sample is immersed in hexane to wash the oil from the exposed surface as a preparation for SEM imaging. This exposes the microstructure but still retains most of the oil in the sample to ensure mechanical stability [25,26,27]. The authors of this review have also found that using ethanol rather than hexane to “wash” the sample is an effective means of preparing oleogels for AFM imaging, Figure 1 and Figure 3 show AFM images obtained using this preparation technique, taken from Ref. [10]. Looking at these images, features are visible over a range of length scales, demonstrating that structure has remained intact during the sample preparation steps [10]. 

**Figure 4:**
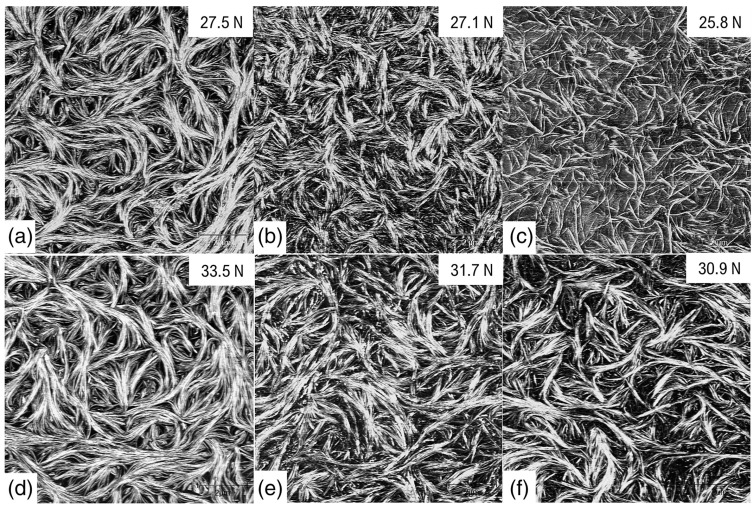
AFM images with a ~10 × 10 μm^2^ field of view for β-sitosterol/γ-oryzanol oleogels in stripped (**a**–**c**) and untreated (**d**–**f**) canola (**a**,**d**), sunflower (**b**,**e**), and flaxseed oil (**c**,**f**). The gel firmness of respective 6% *w*/*w* gel (calculated using a penetration test) is depicted in top right corner. Copyright (2022) Wiley. Used with permission from Scharfe et al. The composition of edible oils modifies β-sitosterol/γ-oryzanol oleogels. Part I: Stripped triglyceride oils, JAOCS, 2022; 99: 43–56.

Scharfe et al. have also used a slightly adapted version of this technique to prepare phytosterol samples for imaging as part of a series of papers investigating how changes to the oil phase alter gel formation and structure [12,13,14]. Some images taken from Ref. [12] are shown in Figure 4. The large fibrous bundles are visible in these images as well as the individual ~10 nm fibrils. Interestingly, they are able to observe how changing the composition of the oil results in differing gel network structure, with far thinner fibre bundles apparent in the stripped flaxseed oil in particular.

**Figure 5:**
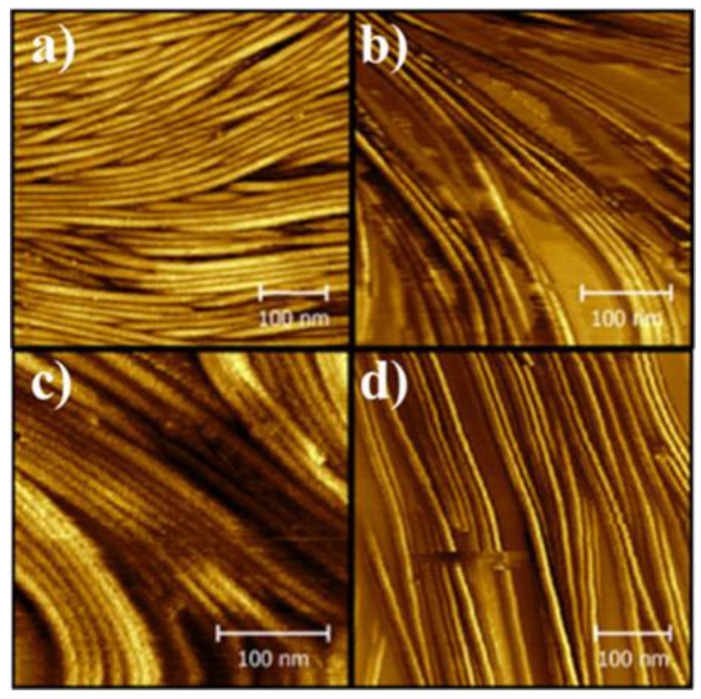
AFM phase images of (**a**) cholesterol and γ-oryzanol, (**b**) cholestanol and γ-oryzanol, (**c**) β-sitosterol and γ-oryzanol, and (**d**) stigmasterol and γ-oryzanol, spincast onto mica. Reprinted (adapted) with permission from Langmuir 2018, 34, 29, 8629–8638. Copyright 2018 American Chemical Society.

Another alternative method to ensure a very thin film of gel with minimal excess oil is to use spincasting, whereby the molten gel is placed on a substrate as it is spun at ≥1000 rpm. Images obtained using this technique were reported for a phytosterol-based gel by Bot and Flöter [15], revealing for the first time the fibrils that make up the gel structure, previously suggested from scattering experiments. This technique was further refined to obtain the images of a range of phytosterol gels shown in Figure 5, taken from Ref. [9]. It is notable that, in Ref. [9], it was profiles taken in the phase mode that provided the best images of the 10 nm tubules (further confirming scattering measurements), demonstrating the value of measuring multiple properties of the sample simultaneously.

**Figure 6:**
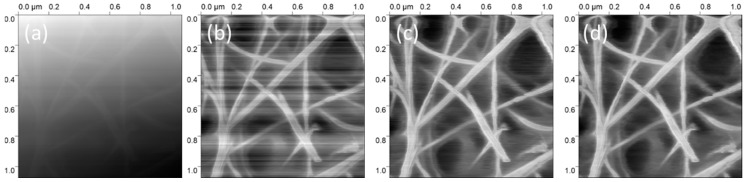
(**a**) Raw AFM height image; (**b**) after polynomial background level correction; (**c**) after row matching; **(d)** after scar correction. Reprinted (adapted) with permission from Langmuir 2017, 33, 18, 4537–4542. Copyright 2017 American Chemical Society.

Dipcasting and spincoating each have benefits and drawbacks, and, as can be seen from Figure 1, Figure 3, Figure 5 and Figure 6, the authors of this review have previously had success imaging oloegels based on phytosterols in triglycerides using both preparation techniques. Spincoating is generally more reliable and may be controlled in a more systematic way by varying the spin speed. However, the very high shearing forces experienced by the gels during their formation can have a strong effect on the resultant structure. In the case of phytosterol gels, it results in fibres not being arranged in the fluid spanning network visible in Figure 1, Figure 3, Figure 4 and Figure 6 and but instead being aligned along the direction of the shearing force, as can be seen clearly in Figure 5 [9,15]. This may not be a problem if one is using AFM as a tool to, e.g., explore the thickness of these individual fibres that form the gel network, but it prevents any information from being gleaned about the broader oleogel structure. Instead, to produce samples that are as representative of quiescently formed gels as possible, the dipcoating process is probably more appropriate [10,14]. 

Once the samples are prepared using the desired technique, images may be obtained. Even with careful preparation, these samples will still contain some unbound triglycerides, making them a challenging system to image. Typically, oleogels are imaged in tapping mode, and it is advisable to start by tapping the sample very gently; the optimal setpoint can vary significantly from sample to sample, so, as a general rule, the best approach is to gradually decrease the amplitude setpoint until features become visible rather than aiming for a specific value. Once features are being picked up with enough detail, scan rates of ~0.1 Hz are advisable to yield good-quality images. 

For the work shown in Figure 1, Figure 3, Figure 5 and Figure 6, Bruker cantilevers (model MPP-11220-10) with nominal spring constant of 40 N/m, resonant frequency of 300 kHz, and tips with nominal tip radius of 8 nm were employed. For the work shown in Figure 4, OLYMPUSOMCL cantilevers (model AC160TS-R30) with nominal spring constant of 26 N/m, resonant frequency of 300 kHz, and a nominal tip radius of 7 nm were used [12]. For the work shown in Figure 2, cantilevers with a resonance frequency of 300 kHz and a nominal tip radius of 10 nm (RTESPA, Bruker) were used. It can therefore be reasonably concluded that, when imaging un-dried triglyceride-based oleogels in tapping mode, cantilevers with resonant frequencies of ~300 kHz, tip radii of ≤10 nm, and spring constants of ~20–40 N/m should be capable of yielding good results. 

Despite tapping mode being the predominant modality for imaging oleogels, some work has been carried out using contact mode. As previously mentioned, by removing the oil from the oleogel and then immersing the resulting xerogel in water and using a fluid cell to overcome the adhesion between the AFM tip and the gelator, it was possible to image ethyl-cellulose gels in contact mode [16]. These measurements employed a Nanoworld PNP-TR triangular cantilever with tip radius 10 nm and a spring constant of 0.08 N/m.

Once images are obtained, they typically must undergo several steps of post-processing. Figure 6 shows a sequence demonstrating how the quality of an image improves following successive processing steps in the software package Gwyddion [28]. 

The first step is to remove the background. To generate Figure 6b, a polynomial background (Limited Total Degree = 2) is subtracted. It was observed that higher degrees do not offer any significant benefit to the flatness of the image, and using higher total degrees of polynomial also risks introducing artefacts to the image, especially if the density of fibrils varies greatly between different parts of the image. Next, Align Rows gets rid of the artefact whereby horizontal bands appear across the image. Gwyddion offers several different means of doing this, but here we found “Matching” offered the best results. Finally, the short horizontal “scars” that appear in the wake of tall features are removed. 

Once images have been adequately processed, quantitative information may be extracted from them. The simplest means for doing this is often just to look at line profiles across various regions of the image. This was done in Refs. [9,10] to confirm the size of phytosterol fibres. However, given that feature sizes may be smaller than the size of the AFM tip itself, care must be taken to not overestimate heights and distances. 

## 3. Complementary and Alternative Techniques to AFM

There are several alternatives to AFM for gaining information on the microstructure of edible oleogels, summarised in Table 1. For a survey of the different imaging techniques available for food systems more generally, we also recommend the comprehensive work of Metelli et al. [29]. For a broader discussion of the techniques that can be used to characterise oleogels at larger length scales, the review by Flöter et al. is also an excellent starting point [30].

The most obvious alternative to AFM imaging for oleogel systems is SEM (scanning electron microscopy) or its low-temperature variant, cryo-SEM. As mentioned previously, with careful sample preparation, Sawalha et al. were able to obtain excellent SEM images that show the fibrous network of phytosterol oleogels [25]. The spatial resolution and imaging capabilities of AFM and SEM are similar, and thus the key consideration may be whether the sample preparation for either technique is more appropriate for the system you wish to image; AFM samples can be easier to prepare as they may be completed at room temperature, do not need deposition of an ultrathin metal coating, supercritical CO_2_, or other chemical processes often used in SEM preparation. However, as outlined previously, the production of thin-film samples for AFM may alter the gel structure in significant ways, most clearly in spincoating. One area where tapping mode AFM has a clear advantage over SEM is the fact that the latter only obtains a single image type, compared to the three different types of plots that may be generated simultaneously by AFM. Cryogenic Transmission Electron Microscopy (cryo-TEM) is another option for looking at nano-scale features in soft-matter samples [31], including in oleogels [32]. Similar to AFM, cryo-TEM requires a very thin section of sample, but, as a cryogenic technique, it requires a rapid cooling to vitrify the sample and prepare it for imaging, somewhat complicating the sample preparation. Whether AFM, SEM, or TEM are more appropriate for the oleogel system you wish to study will depend on the details of its structure and the specific focus of the investigation, and (if available) it may be wise to initially employ all three approaches until it is clear which one will be most worth proceeding with for detailed studies.

As well as scanning probe microscopy, there are a suite of super-resolution optical microscopy techniques, such as stimulated emission depletion (STED) microscopy, which may prove useful for imaging sub-diffraction limit features [33]. However, it should be noted that these techniques depend upon structures exhibiting the correct spectroscopic qualities such that fluorescence may be excited by a laser source. In phytosterols, for instance, the absorbance features are at <320 nm [9], which will preclude excitation by anything except a UV source, and there may be a very high fluorescent background due to auto-fluorescence of organic compounds in the oil. One way round this may be to add a fluorescent label to the gelator, but, in systems where a very fine balance of competing physico-chemical interactions dictate the self-assembly processes, care would have to be taken to ensure that addition of these labels did not alter the gel structure. Despite these drawbacks, optical microscopy techniques do benefit from not requiring the removal of the oil phase, and the fact that (particularly if a confocal system is employed) the gel may be imaged at a depth of several micrometres into the sample, rather than just on the surface [23]. 

For those edible oleogel systems where typical feature sizes are larger than a few hundred nanometres, standard brightfield microscopy is often the easiest solution. For looking at larger crystalline features in gels based on fatty crystals, polarised microscopy has proven to be highly effective at enhancing contrast between the liquid gel and structurant [34]. Although the fibres that constitute the phytosterol oleogel network are sub-diffraction limit, emulsions based on these materials can also be imaged with either brightfield or fluorescence microscopy (with dye added to the aqueous phase) [23]. 

To generate a spatial map of mechanical properties (similar to an AFM phase map), an alternative method is particle tracking microrheology. Microrheology is a technique whereby colloidal beads are embedded in the sample and the motion of the bead under Brownian motion is used to discern the local visco-elastic properties of the sample [35]. This may be passive, where beads are observed freely undergoing thermal motion, or active microrheology, where an optical trap is used to constrain the bead. The spatial resolution of this technique is typically on the scale of microns, much larger than the nano-scale resolution probed by AFM, but it does allow for a full 3D viscosity map to be developed and may provide information on the anisotropic viscoelastic response of the material in different directions [36].

As well as both scanning probe and optical microscopy, scattering techniques can provide a great deal of information on the typical length scale of the gel structures. For instance, small-angle X-ray scattering (SAXS) and small-angle neutron scattering (SANS) were able to reveal significant information regarding the structure of phytosterol oleogels before AFM or SEM images could be obtained [11,37]. As well as SAXS and SANS, benchtop scattering techniques such as dynamic light scattering (DLS) may also be effective in oleogels where the structure is due to vesicles or inverse micelles rather than a more brittle fibrous network [24]. The caveat is that, although these scattering techniques provide a measurement of the typical feature size of the gel structure, they are by their definition not direct-imaging techniques and provide only bulk-averaged measurements and thus cannot provide information as to local topography and heterogeneity of the micro/nano-scale structure.

AFM is a powerful tool for providing insight into the gel network structure, but, in common with other imaging techniques, it does not provide information about the molecular interactions that underpin the formation of these structures. It is, therefore, useful to combine AFM with spectroscopic techniques such as UV–Vis absorption, circular-dichroism (CD), Raman, or FTIR [9,38,39]. For absorbance techniques, due to the high concentration of the gelator in typical oleogels (e.g., >5% for phytosterol gels), thin films are necessary to avoid saturation of the absorption peaks probed by these techniques. Therefore, the sample preparation techniques outlined for AFM measurements are also relevant to FTIR, UV–Vis, and CD spectroscopy of phytosterol oleogels (with a substrate that is transparent in the relevant spectral window replacing the mica sheet) [9]. For scattering techniques such as Raman, this is not necessary. Instead, the limiting factor will more likely be the endogenous fluorescent background, which may overwhelm the Raman signal. In Ref. [9], this problem was encountered, and, to reduce the antagonistic fluorescence signal, the sample was exposed to laser excitation for approximately 20 min to photo-bleach endogenous chromophores before Raman spectra were measured.

As well as the final structure, it is often of interest to researchers to understand the formation kinetics of oleogels. AFM is less suitable for studying nucleation kinetics in the early stages of self-assembly before gelation occurs as a solid sample is needed for high-resolution imaging. Additionally, AFM has a long acquisition time relative to most other techniques, and it is often difficult to arrest the gelation process midway in a manner that allows AFM measurements to be carried out. Instead, super-resolution imaging, and particularly stimulated emission depletion (STED) microscopy [33], cryo-TEM [31], and NMR [40], have been successful in elucidating nucleation and growth and self-sorting mechanisms for polymer fibril hydrogels. It is highly likely that these methods can be extended to oleogelators, with progress already having been made in the application of NMR to oleogels [41].

## 4. Future Outlook

It is worth considering some possible future uses of AFM in imaging edible oleogel systems. One particularly interesting future use of AFM would be to perform force mapping measurements and measure the mechanical properties of the fibres that make up the gel network in situ. This sort of study has already been extensively performed to characterise the properties of polymeric hydrogels [42,43]. For many oleogels (including the phytosterol–triglyceride gels that are the focus of this work), the bulk gel properties are well characterised using rheological tools, and there is a clear understanding of the microstructure of the gel network, but relatively little is known about the mechanical properties of the individual fibrils that make up the gel network. This has ramifications for the design of novel gelator molecules; for instance, knowledge of whether inter-fibril or intra-fibril bonds are more likely to yield first would be instructive. Recently, more advanced force mapping modes, such as PeakForce Tapping [44,45] and Quantitative Imaging (QI mode) [46,47], have been introduced. They are superior to traditional “force mapping” as they allow the high-resolution topography imaging of material surfaces while collecting simultaneously high-resolution images of adhesion, stiffness, and moduli. PeakForce Tapping is based on sinusoidal tapping of the sample with a force controlled by a feedback loop. QI mode uses force–distance curves at a high rate with direct force control. Owing to their superior control of the applied forces (which can be very low at the range <10 pN), they work particularly well with soft/fragile samples. 

As mentioned above, AFM imaging of the oleogel formation process in a triglyceride would be highly challenging. However, this has been performed for oleogels based upon solvents such as octanol where 12 × 12 μm^2^ images were obtained at rates of ~0.3 min^−1^ [48], but, to the best of our knowledge, it has yet to be performed for gels based on triglycerides. The ability to see gels form in real time would be highly beneficial to both a fundamental understanding of the physico-chemical processes underpinning gelation and a better knowledge of how to incorporate these gels into food systems. A prerequisite of any such system would be excellent environmental control of the AFM and fast enough scan speeds such that image acquisition occurs on a shorter time scale than the gelation process.

Finally, as previously outlined, there are various benefits and drawbacks of AFM vs other imaging modalities. However, the combination of AFM with advanced optical microscopy is particularly promising [49,50,51,52,53]. It would be highly interesting to use AFM in conjunction with fluorescence or Raman imaging modalities simultaneously to observe spectroscopic signatures associated with gelation on the same field of view as that of the AFM image. 

## 5. Conclusions

AFM imaging is a powerful technique for obtaining detailed information of the gel structure, as well as visually appealing images of oleogels. It is, however, not the only imaging modality available, and it may prove to be most valuable when used in concert with other complementary techniques. Scattering and spectroscopic techniques in particular may prove useful in relating how the microstructure revealed by AFM functions stems from molecular interactions and stacking motifs. It is also prudent, if possible, to compare the AFM results to those obtained via other imaging techniques, whether they are optical (i.e., confocal or super-resolution microscopy) or scanning probe (i.e., SEM or TEM) to see if one of these modalities offers additional information. Equally, the quality of AFM images of oleogels depends largely on the preparation of samples that are thin enough to be imaged and where the AFM tip can interact with the features rather than just a deep layer of oil. One must thus weigh the advantages and disadvantages of different preparation techniques depending on the length scale and feature size they wish to explore. To this end, the authors hope this review of AFM imaging of oleogels is a useful introductory guide.

## Figures and Tables

**Table 1:** Comparison of AFM and other imaging and analysis protocols.

Technique	Length Scale of Features Resolved	Sample Preparation Required	Information Obtained
AFM	~1 nm	Thin film	High-resolution map of topography and material properties.
Cryo-SEM	~1 nm	Gel must be frozen at cryogenic temperature	High-resolution 2D images.
Cryo-TEM	~1 nm	Thin film; gel must be frozen at cryogenic temperature	High-resolution 2D images.
Widefield microscopy	~200 nm	Bulk sample	2D images.
Confocal laser scanning microscopy	~200 nm	Sample must be labelled with appropriate fluorescent probe.	3D image stacks.
Super-resolution microscopy	~ 50 nm	Sample must be labelled with appropriate fluorescent probe.	High-resolution images.
SAXS	~1 nm (Bulk, not spatially resolved)	Bulk sample	Typical length scale and shape of structures in sample.
SANS	~1 nm (Bulk, not spatially resolved)	Bulk sample, deuterated solvents needed	Typical length scale and shape of structures in sample.
Microrheology	~1 µm	Dilute solution of tracer beads placed in sol.	Low-resolution map of material properties.

## Data Availability

No new data was generated for this article.
